# Case report: A severe case of generalized tetanus in a young cat

**DOI:** 10.3389/fvets.2023.1328331

**Published:** 2023-12-07

**Authors:** Jessica Zilli, Thomas C. Häußler, Edward J. Ives, Martin J. Schmidt

**Affiliations:** ^1^Department of Veterinary Clinical Sciences, Small Animal Clinic – Clinical Neurology, Neuroradiology and Neurosurgery, Justus-Liebig-University, Giessen, Germany; ^2^Neurology and Neurosurgery Service, Anderson Moores Veterinary Specialists, Linnaeus Veterinary Limited, Winchester, United Kingdom; ^3^Department of Veterinary Clinical Sciences, Small Animal Clinic – Surgery, Justus-Liebig-University, Giessen, Germany

**Keywords:** amputation, *Clostridium tetani*, extensor rigidity, feline, necrotizing fasciitis

## Abstract

A 10-month-old, 3 kg, female spayed Domestic Shorthair cat was presented with a chronic, infected wound at the level of the proximo-lateral left pelvic limb. General physical examination revealed a weight-bearing lameness of the left pelvic limb, which was moderately and circumferentially swollen and edematous proximal to the tarsal joint. On the lateral aspect of the proximal thigh, there was a chronic wound of 1 cm in diameter and an additional exudative skin lesion was present throughout the whole length of the caudo-lateral thigh. Complete blood count and serum biochemistry profile revealed mild anemia, increased serum amyloid A, hyponatraemia, hypochloraemia, hypocalcaemia, hyperkalaemia, hypermagnesaemia, hyperglycaemia, increased creatine kinase, and increased liver parameters. Surgical exploration of the wound was performed, and necrotizing fasciitis was suspected. The affected limb was amputated and swabs for bacterial culture were taken from both the skin lesions and surgical site before wound closure. One day after surgery, mild muscular contractions on the forehead and an increased muscle tone of the right pelvic limb were evident. One day later, the cat developed a generalized increase in extensor tone, with intermittent opisthotonos, resulting in lateral recumbency. Based on these clinical signs, a diagnosis of generalized tetanus was made and treatment with midazolam, methocarbamol, and metronidazole was started. Despite an improvement of all blood parameters, the cat progressively deteriorated and 4 days after surgery, it developed episodes of tetanic convulsions, associated with hyperthermia and ventricular arrhythmias. Despite intensive care and medical management, the cat died following a cardio-respiratory arrest 3 days later. This case report describes a rare case of severe generalized tetanus in a young cat.

## 1 Introduction

Tetanus is a neurological disorder caused by the toxin tetanospasmin, which is produced by the vegetative form of the Gram-negative, motile, spore-forming, anaerobic bacillus *Clostridium tetani*. Bacterial spores usually penetrate through a wound in the skin or mucosal membranes. Under anaerobic conditions, spores germinate to become the vegetative form of the bacterium which is capable of toxin production (1). From the infection site, tetanospasmin reaches local terminations of peripheral motor neurons, with retrograde axonal transport taking the toxin towards the central nervous system (CNS) where it interferes with the release of inhibitory neurotransmitters glycine and gamma aminobutyric acid (GABA) in the brainstem and spinal cord (2, 3). The consequence of this central disinhibition is sustained and uncontrolled skeletal muscle contraction. Autonomic signs may also be evident in more severely affected individuals (1).

*Clostridium tetani* spores are ubiquitous in the environment, but infections and disease development are uncommon in dogs and very rare in cats. In comparison to humans, horses, mice and guinea pigs, both dogs and cats are relatively resistant to the effects of the toxin, with cats in particular known to be more resistant than dogs (1, 4, 5). Although the generalized form of tetanus is well described in dogs, cats are typically affected by the localized form and signs are usually less severe than in dogs. In addition, disease onset is usually delayed in cats, and may take up to 3 weeks after infection, compared to within 5 to 10 days in dogs (range: 3–18 days) (1, 5).

This case report describes a severe case of generalized tetanus in a Domestic Shorthair cat. The disease progression in this case was rapid, and the cat died within 1 week after onset of the first clinical signs. The cause of death was cardiorespiratory arrest as a consequence of cardiac arrhythmias and severe muscular contractions, with likely secondary paralysis of the respiratory muscles.

## 2 Case presentation

**Figure 1:**
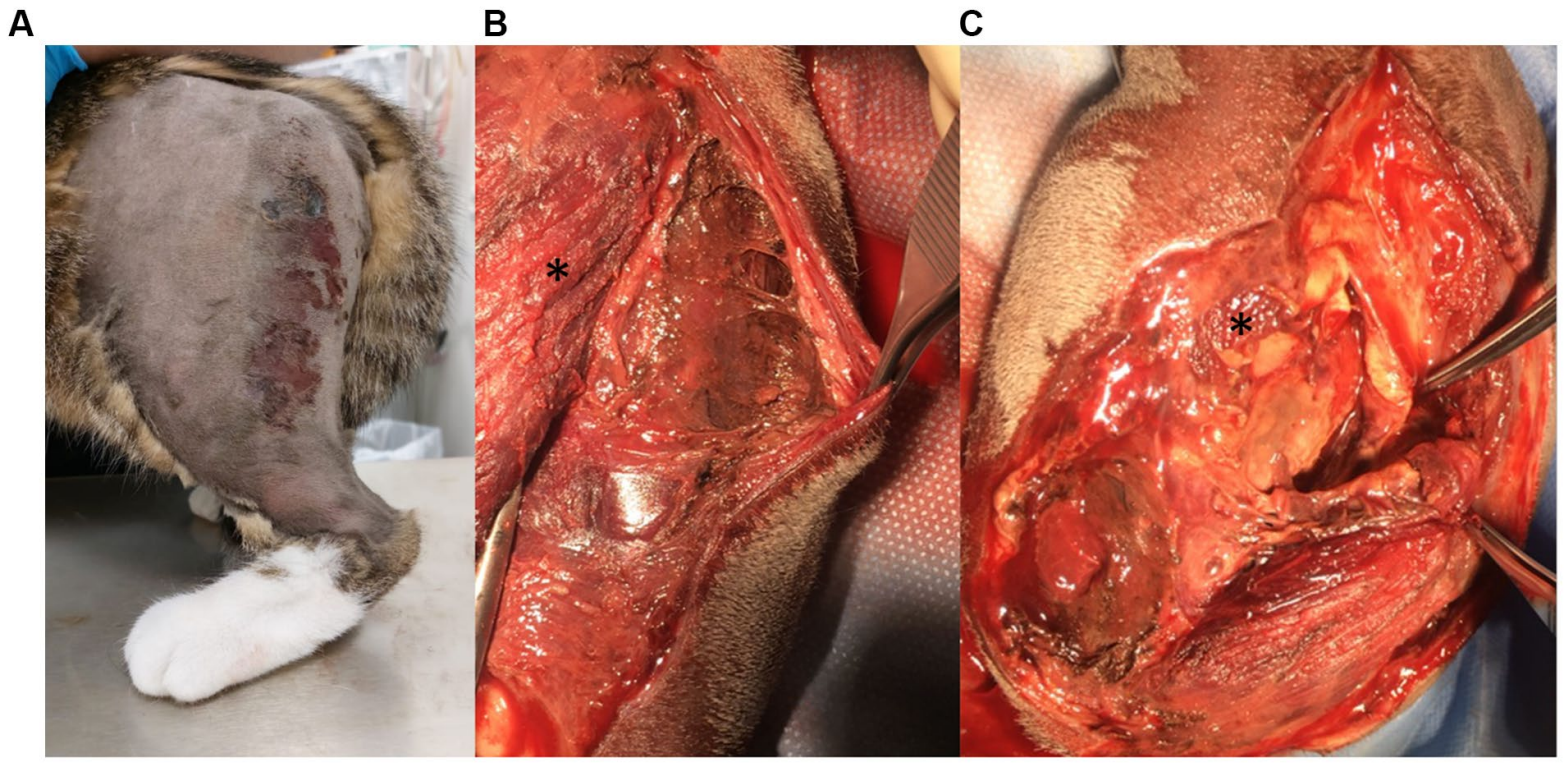
Preoperative view of the left pelvic limb **(A)** and intraoperative view of the left thigh **(B,C)**. **(A)** Lateral to the proximal femoral epiphysis, a chronic, perforating wound could be seen and distal to this, an exudative skin lesion was present; this extended proximo-distally from the wound to the level of the stifle joint, was *circa* 3–4 cm wide and was located over the latero-caudal aspect of the thigh. The paw was circularly edematous. **(B,C)** Intraoperatively, there were severe necrotic changes affecting the muscles of the lateral thigh, including the *m. vastus lateralis* (*) and the *m. biceps femoris*, and the surrounding fat and connective tissues, with palpable subcutaneous emphysema.

A 10-month-old, 3 kg, female spayed Domestic Shorthair cat was presented to the surgical department of the Small Animal Clinic, Justus Liebig University in Giessen with a history of lethargy and a chronic, infected wound of the left pelvic limb. Three days prior to referral, the cat was found after being away for 2 days; the cause of the wound was unknown. A rectal temperature of 43°C was recorded on first presentation at the referring vet, who had initiated treatment with an unknown anti-inflammatory drug (likely meloxicam) and an unknown antibiotic prior to referral. Except for the lethargy and the left pelvic limb abnormalities, general physical examination was normal. The left pelvic limb was circumferentially, moderately swollen and edematous proximal to the tarsal joint. On the lateral aspect of the proximal left thigh, there was a chronic wound with a diameter of 1 cm and an additional exudative skin lesion along the whole length of the caudo-lateral aspect of the thigh (Figure 1A). In the left inguinal area, there were multiple, minor lacerations of the skin. The cat showed a moderate weight-bearing lameness [grade 2/4 according to Brunnberg et al. (6)] of the left pelvic limb. Severe discomfort was apparent during examination of the limb, and subcutaneous emphysema was evident around the affected area. The complete blood count showed a mild, normocytic, normochromic, non-regenerative anemia with a hematocrit of 0.3 L/L (range: 0.31–0.52). Plasma biochemistry profile revealed multiple abnormalities, including electrolyte derangements, increased hepatic parameters and a marked increase of the creatine kinase and Serum Amyloid A (SAA) (Table 1). Cytologic evaluation of a blood smear revealed severe toxic changes of the neutrophils.

**Table 1:** Abnormal biochemistry parameters on the day of presentation and on recheck, 4 days after surgery.

Parameter	Value on presentation	Recheck value	Unit of measurement	Range
Sodium	143	153	mmol/L	150–163
Chloride	100	111	mmol/L	108–121
Potassium	6.05	4.55	mmol/L	3.6–4.8
Ionized calcium	1.07	1.26	mmol/L	1.19–1.41
Phosphate	2.13	2.15	mmol/L	0.8–1.9
Magnesium	0.93	0.6	mmol/ml	0.3–0.55
Glucose	19.1	11.5	mmol/L	3.89–6.11
Bilirubin	72.79	3.66	μmol/L	0–3.4
Alanine aminotransferase	340	123	U/L	0–70
Glutamate dehydrogenase	32	3	U/L	0–11.3
Creatine kinase	344,400	2,289	U/L	<205
Serum Amyloid A	174.7	43.8	μg/L	<3.9

The values marked in blue are below the reference range, and those marked in red above it.

Thoracic and abdominal radiographs were unremarkable. Radiographs of the left pelvic limb were normal other than severe subcutaneous emphysema of the left proximal thigh.

Surgical wound debridement was elected and, after initial stabilization with isotonic saline (2 mL/kg/h; Isotone Kochsalz-Lösung 0.9%, B.Braun Vet Care, Melsungen, Germany) fluid therapy, the cat was premedicated with midazolam (0.2 mg/kg; Midazolam-ratiopharm, Ulm, Germany) and ketamine (5 mg/kg IV; Ketamine 10%, Serumwerk Bernburg, Bernburg, Germany). Anaesthesia was induced with propofol (2 mg/kg, IV; Proposure, WDT, Garbsen, Germany), and maintained with isoflurane (IsoFlo 100%; Ecuphar, Greifswald, Germany) and fentanyl (20 μg/kg/h, IV; Fentadon 50 μg/mL, Dechra, Aulendorf, Germany). The cat was also administered noradrenaline (0.2–2 μg/kg/min, IV; Arterenol, Cheplapharm, Greifswald, Germany) for vasopressor support due to hypotension, fluid therapy using a crystalloid solution (5 mL/kg/h, IV; Sterofundin ISO, B.Braun Vet Care, Melsungen, Germany) and amoxicillin-clavulanic acid (20 mg/kg, IV q8h; Amoxiclav Hexal i.v. 500/100 mg, Holzkirchen, Germany). Intraoperatively, the musculature of the thigh was necrotic and multiple vessels were thrombotic (Figures 1B,C). Therefore, the limb was amputated by exarticulation at the level of the hip joint. Two closed suction drainage systems were placed and culture swabs from the skin lesions and the surgical site were obtained before skin closure.

**Figure 2:**
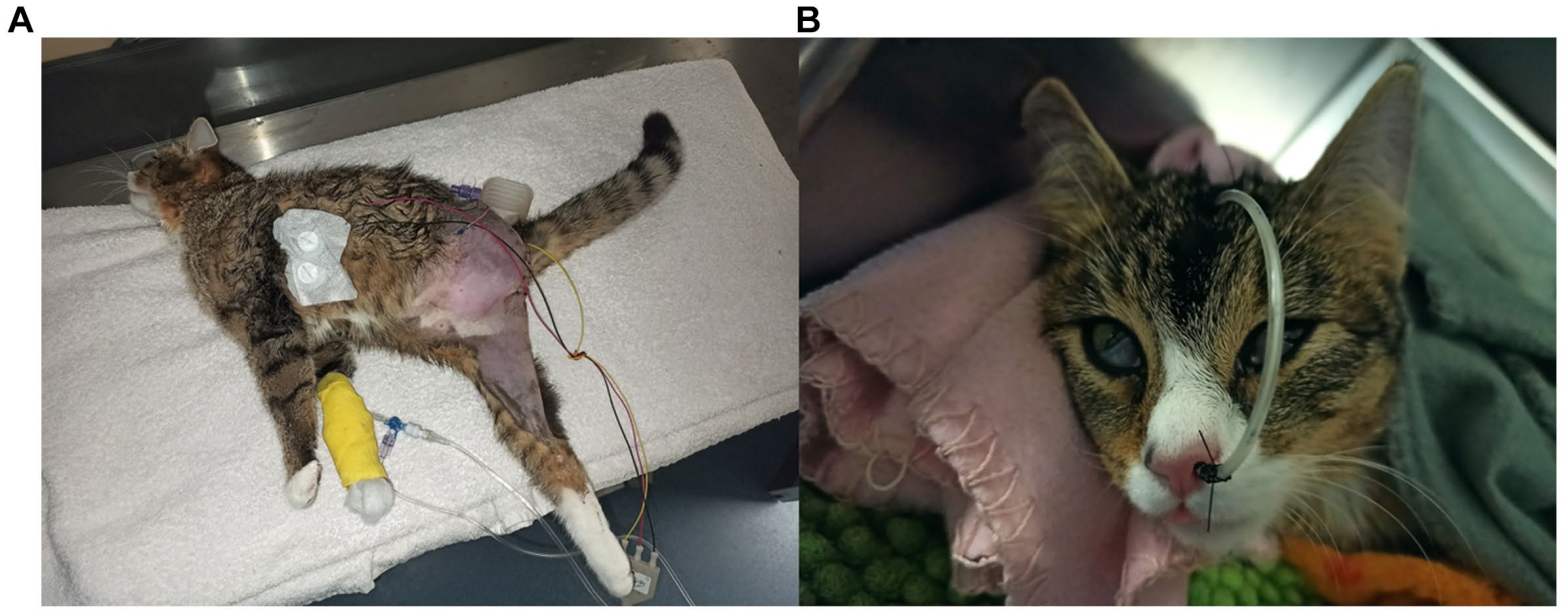
Ten-month-old, female neutered domestic shorthair cat 2 days **(A)** and 4 days **(B)** after amputation of the left pelvic limb due to a necrotizing fasciitis and myositis. The cat shows severe generalized extensor rigidity, with opisthotonos and increased extensor tone of the tail **(A)**; the facial musculature is also mildly contracted. Subsequently, it developed stiffness of both ears and bilateral protrusion of the third eyelids **(B)**, as a consequence of the tetanus toxin effect on the extraocular muscles which leads to enophthalmos (5).

Postoperative pain was managed with a fentanyl constant rate infusion at a dosage of 5 μg/kg/h. The cat was mildly depressed, inappetent, tachycardic (160–200 /minute) and hypotensive (80–110 mmHg), without any neurological abnormalities at that time. Due to the severity of the infection, marbofloxacin (2 mg/kg, IV q24h; Marbocyl 1%, Vetoquinol, Ismaning, Germany) was added to on-going antibiotic treatment with amoxicillin-clavulanic acid. Physiological blood pressure was maintained with noradrenaline (0.2 μg/kg/min, IV). A central venous catheter was placed after surgery and a naso-esophageal tube allowed early feeding. One day after surgery, the cat’s forehead muscles appeared contracted, and the muscle tone of the right pelvic limb was mildly increased. Two days after surgery, the neurological abnormalities became more evident; the cat was increasingly obtunded, it started being very sensitive to light and loud noises, and it developed a generalized increase in extensor muscle tone, with intermittent opisthotonos (Figure 2A). The extensor tone of the right pelvic limb and tail were markedly increased, with hyperextension of the right tarsal joint, and both thoracic limbs were also increased in tone, with worsening of the muscular contractions when the cat was manipulated. Due to the increased muscle tone, the spinal reflexes of the three remaining limbs could not be elicited. An intermittent protrusion of the third eyelids was also observed and the facial muscles were mildly contracted. Differential diagnoses for an acute onset of such neurological signs included “stiff-man syndrome,” pseudomyotonia or myotonia, strychnine poisoning, hypocalcaemia and meningitis. However, in view of the characteristic, progressive clinical signs and clinical history, a diagnosis of generalized tetanus class III (7) secondary to necrotizing fasciitis was considered most likely. The owner declined electromyography to further characterize the condition.

The cat showed an initial, mild improvement directly after administration of midazolam (0.5 mg/kg, IV) and methocarbamol (20 mg/kg, IV; Ortoton K.I.S., Recordati Pharma, Ulm, Germany). Further therapy included a continuous rate infusion (CRI) of midazolam (0.5 mg/kg/h, IV) and methocarbamol (10 mg/kg/h, IV), and application of a tear substitute (q6hr, Hylo Comod, Ursapharm, Saarbrücken, Germany). Treatment with metronidazole (25 mg/kg, IV q12h; Metronidazol, deltamedica, Reutlingen, Germany) was started and marbofloxacin was discontinued (5). Amoxicillin-clavulanic acid and noradrenalin were continued at the same dosage, fentanyl was substituted with buprenorphine (0.1 mg/kg, IV q8h; Buprenovet multidose, Richter Pharma AG, Wels, Austria), and meloxicam was started (0.05 mg/kg, PO q24h; Metacam 0.5 mg/mL, Boehringer, Ingelheim, Germany). The cat was kept in a calm and dark environment and the cat’s recumbency was changed every 4 hours to avoid ventilation problems and pressure sores.

Despite intensive treatment and a significant improvement of the blood parameters (Table 1), the cat progressively deteriorated. At this stage, her tetanus severity grading increased to class VI (7) and 4 days after surgery, the cat developed sudden episodes of tetanic convulsions, usually associated with hyperthermia and ventricular arrhythmias. These episodes could be interrupted by the administration of midazolam boli (0.5 mg/kg, IV). Repeated neurological examinations revealed on-going obtundation, erect and stiff ears, contraction of the facial muscles, permanent protrusion of the third eyelids (Figure 2B), and unchanged moderate to severe extensor rigidity of all three limbs and the tail. A further skeletal muscle relaxant, dantrolene (2 mg/kg, PO q8h; Dantamacrin 25 mg, Norgine, Wettenberg, Germany), was introduced (via the naso-esophageal tube) and the cat was also started on dexmedetomidine (0.5–2 μg/kg/h, IV; Dexdomitor, Vetoquinol, Espoo, Finland). A higher dose of dexmedetomidine was avoided due to the development of hypothermia in-between tetanic convulsions. Moreover, although urination was still maintained, a urinary catheter was placed to facilitate the management of the patient. The results of the cultures from the skin lesion revealed the presence of *Clostridium perfringens* and *Pseudescherichia vulneris*. The surgical site cultures sampled before skin closure were negative. Despite continued intensive therapy, the episodes of tetanic convulsions did not improve, and the cat developed a cardio-respiratory arrest 7 days after surgery. The owners declined cardio-pulmonary resuscitation. Post-mortem examination was not performed given the owner’s financial concerns.

## 3 Discussion

Generalized tetanus is a rare presentation in cats and there are only sporadic case reports in the literature (8–15). Cats are known to be 2000 to 7,200 times more resistant to tetanus than horses due to the difficulty of the toxin to penetrate the central nervous system and bind to the appropriate receptors (1, 4, 11). For this reason, the disease most commonly manifests in cats as a localized form affecting one or two limbs, with the affected muscle groups usually close to the source of toxin (i.e., wound and surgical site) (12, 16–18). A localized form of tetanus has also been described in queens after ovariohysterectomy; those cats showed involvement of the lumbar muscles and sometimes one or both pelvic limbs (19). In comparison, the cat in this report had a severe progression of clinical signs to a generalized form with whole body muscular contractions and additional autonomic signs (i.e., cardiac arrhythmias). Autonomic nervous system involvement is associated with a worse prognosis in humans and dogs with tetanus, and in people can be unrelated to the severity of neuromuscular signs (7, 20). It has also been reported that younger animals may develop a more severe form of the disease, as for the cat in this report (7, 21). This is potentially explained by the development of an acquired immunity against the tetanus toxin in adults after repeated environmental exposure to its antigens (1, 7, 22). Other prognostic factors have been described for dogs with generalized tetanus and, if these factors are extrapolated to feline cases, the cat of this report presented multiple negative prognostic factors, such as an age < 2 years old, the severity of the clinical signs (class III to VI), short duration of disease-specific signs before recognition of the diagnosis, and occurrence of hyperthermia (7, 21).

Another factor which may have contributed to the outcome in this case was the advanced stage of the soft tissue trauma and the concurrent systemic disease observed. A diagnosis of a necrotizing fasciitis was made following surgery, with the development of tetanus 2 days after presentation and subsequent death due to a cardiopulmonary arrest 5 days later. Acute cardiopulmonary arrest in tetanus patients may be the consequence of respiratory muscle paralysis and/or cardiac dysrhythmias. Nevertheless, it is also important to consider that the outcome in cats with vasoplegic shock compared to the other two phenotypes of septic shock (cryptic and dysoxic) is worse, with a reported mortality rate of 91% (23).

Necrotizing fasciitis is a rapidly progressive infection of the subcutaneous tissues, extending into the fascia and occasionally also involving the underlying muscles (24). It has been most commonly reported in association with Streptococcus species infections (25). However, the same clinical findings have been also described with other bacterial infections, including *Staphylococcus pseudintermedius, Staphylococcus aureus, Pasteurella multocida, Escherichia coli* and *Serratia marcescens* (24). Necrotizing fasciitis has been reported in association with various clostridium species infection in humans (e.g., *C. difficile*, *C. perfringens*, *C. tertium* and *C. septicum*), therefore, it can be hypothesized that similar changes may also occur secondary to clostridial infection in dogs and cats (26–28). Secondary *Clostridium tetani* infection of a primary fasciitis caused by another bacterial species is equally possible in this case.

Tetanus antitoxin, which aims to neutralize unbound tetanospasmin, was initially not administered in the cat of this report due to the well-known tetanospasmin resistance of this species (1) and to the risk for anaphylactic shock even in animals that do not react to subcutaneous/intradermal injection prior to intravenous administration (5). Studies have also underlined the importance of timing for the administration of antitoxin, which should be ideally injected before surgical debridement of the infected wound (12, 16). In this regard, another reason for which the administration of antitoxin was not considered in this case was that surgical treatment of the wound (which was suspected to be the source of tetanospasmin) had already been performed at the time the cat developed neurological signs. However, in cases with fulminant disease progression, administration of tetanus antitoxin might improve the prognosis, as suggested in previous reports, and it is therefore possible that early administration at the time of admission, or when the neurological signs were first recognized after surgical treatment, could have improved the outcome in this case (14, 17). Nevertheless, it is still unclear whether the administration of antitoxin influences the clinical course of the disease, and this should be evaluated in future studies on cats with generalized tetanus (7, 12, 21).

In dogs and cats that present with tetanic signs, wounds are frequently small or may not even be found (7, 11, 12, 14, 16–18, 29). However, it has been suggested previously that the underlying cause can be usually identified in cats compared to dogs (12). In the cat of this report, the extent of the soft tissue infection was severe but not recognized by the referring veterinarian due to lack of wound exploration and debridement. This led to delayed wound treatment which was potentially associated with a worse prognosis. Although no studies have extensively examined the survival rate in cats with tetanus so far, according to the current literature the overall prognosis appears to be good if clinical signs remain focal and/or can be medically controlled (12, 16). Nevertheless, it is important to mention that as soon as cats develop signs of generalized tetanus, particularly when associated with autonomic involvement, the prognosis is likely to be as guarded as for other species (1, 21). It has been also suggested that the shorter the incubation period, the worse the clinical progression and the prognosis (1, 19). This was consistent with the cat of this report, in which the muscular rigidity began *circa* 5–6 days after the wound had been first noticed.

The diagnosis of tetanus could not be confirmed through the isolation of *C. tetani* in this case. Indeed, only *C. perfringens* was isolated. However, this is a common scenario in tetanus cases, which tends to be a clinical diagnosis, as bacterial cultures often lead to false negative results and a positive result may not automatically imply toxin production (5). Due to the clinical history and fulminant progression, as well as the presence of another Clostridium species in the wound, tetanus was still considered the most likely differential diagnosis. Other possible differentials were “stiff-man syndrome” (considered unlikely since it has only been described in dogs and horses so far, in addition to its episodic signs) (30, 31), pseudomyotonia or myotonia (unlikely due to progression and absence of phases with muscular relaxation), strychnine poisoning (excluded since the clinical signs started in the hospital where the animal did not have access to this substance, and the clinical progression was considered to be too slow), hypocalcemia (excluded with the biochemistry), and meningitis. Unfortunately, post-mortem examination was not performed. However, this may have still not provided a final diagnosis given that the cat received antibiotic treatment before death.

In regard to the management of tetanus in this case, sedation with dexmedetomidine may help to provide anxiolysis, muscle relaxation and control of autonomic signs (15). In the current case, the cat showed a clinical improvement after administration of dexmedetomidine; nevertheless, it was still experiencing episodes of tetanic seizures, hyperthermia and arrhythmias even when administered at higher doses (2 μg/kg/h, IV). In addition to dexmedetomidine, three other muscle relaxants were administered in this case: midazolam, methocarbamol and dantrolene. In previous feline reports, the efficacy of methocarbamol and dantrolene was considered questionable (18, 19). Nevertheless, methocarbamol appeared to show good efficacy when used in combination with midazolam at the start of therapy in this case. The use of dantrolene has been reported in human patients with tetanus, as it inhibits Ca^+2^ release from the sarcoplasmic reticulum (32). For the current case, it is difficult to assess to what degree dantrolene contributed to muscle relaxation, since the cat was already receiving three further myorelaxants. Magnesium supplementation has been reported to be helpful in the management of severe muscle contractions in 3 dogs with tetanus (33, 34). However, a recent study did not report a difference in survival for dogs receiving magnesium and those that did not, and it was not used in the present case (21).

In conclusion, this case report describes a rare case of generalized tetanus in a cat secondary to a severe soft tissue infection of the left thigh. Despite intensive care, sedation and treatment with up to four different myorelaxants, the cat was still showing tetanic convulsions and eventually died during one of these episodes. It is unclear whether the administration of antitoxin, magnesium or other medications (e.g., phenobarbitone) would have changed the outcome in this cat. Due to lack of information regarding tetanus in cats, particularly generalized cases, future studies are necessary to evaluate the most effective treatment, outcome and prognosis.

## Data availability statement

The original contributions presented in the study are included in the article/supplementary material, further inquiries can be directed to the corresponding author.

## Ethics statement

Ethical approval was not required for the studies involving animals in accordance with the local legislation and institutional requirements because this was a retrospective single animal case report rather than research. Written informed consent was obtained from the owners for the participation of their animals in this study. Written informed consent was obtained from the owners for the publication of this case report.

## Author contributions

JZ: Conceptualization, Writing – original draft, Writing – review & editing, Investigation. TH: Supervision, Writing – review & editing, Investigation. EI: Writing – review & editing, Supervision. MS: Funding acquisition, Writing – review & editing, Supervision.
